# Case report: revision surgery for a fractured femoral stem using an ESOP prosthesis – analysis of the diagnostic work-up, treatment strategy, and long-term outcome

**DOI:** 10.1093/jscr/rjag958

**Published:** 2026-10-22

**Authors:** Łukasz Dutka, Julian Dutka, Adrian Szczecina, Adam Solarz, Dominik Kostarczyk, Mateusz Bukowczan

**Affiliations:** Department of Orthopedic and Trauma Surgery, Żeromski Specialist Hospital in Kraków, Osiedle Na Skarpie 66, 31-913 Kraków, Poland; Department of Orthopedic and Trauma Surgery, Stefan Żeromski Specialist Hospital in Kraków, Osiedle Na Skarpie 66, 31-913 Kraków, Poland; Department of Orthopedic and Trauma Surgery, Stefan Żeromski Specialist Hospital in Kraków, Osiedle Na Skarpie 66, 31-913 Kraków, Poland; Department of Trauma and Orthopedic Surgery, Multispecialty Hospital in Jaworzno, Ul. Chełmońskiego 28, 43-600 Jaworzno, Poland; Department of Orthopedic and Trauma Surgery, Stefan Żeromski Specialist Hospital in Kraków, Osiedle Na Skarpie 66, 31-913 Kraków, Poland; Department of Orthopedic and Trauma Surgery, Stefan Żeromski Specialist Hospital in Kraków, Osiedle Na Skarpie 66, 31-913 Kraków, Poland

**Keywords:** total hip arthroplasty, femoral stem/implant fracture, DiaLoc/Zweymüller, modular stem, short-stem hip prosthesis, stress shielding

## Abstract

Femoral stem fracture following total hip arthroplasty is a rare but serious complication that usually requires removal of both implant fragments and implantation of a longer revision stem. However, this procedure may be associated with substantial bone loss and an increased risk of intraoperative and postoperative complications. A 58-year-old man underwent cementless total hip arthroplasty with a Zweymüller-type stem in 2008. Five years later, the stem fractured. During revision surgery, the proximal fragment was removed, whereas the well-fixed distal fragment could not be safely extracted. It was therefore retained, and only the proximal component of a modular ESOP (anatomical modular cementless femoral stem) short stem was implanted. The postoperative course was uneventful. At 13-year follow-up, the patient remains pain-free, with full hip function, and no radiographic evidence of implant loosening. In carefully selected cases, this technique may represent a valuable alternative to extensive revision surgery.

## Introduction

Total hip arthroplasty (THA) provides implant survivorship of ~95%. Femoral stem fracture is nevertheless a rare but serious complication, reported in ˂0.1%–3.4% of cases, and its treatment may require extensive revision surgery.

The DIALOC® cementless femoral stem is manufactured from titanium–aluminum–niobium alloy. Its tapered rectangular design and slight medial curvature provide axial and rotational stability through metaphyseal and diaphyseal fixation, while the porous surface promotes osseointegration and load transfer along the implant [1].

Stem fracture is usually related to fatigue failure caused by repetitive loading and abnormal load distribution. Inadequate proximal support with persistent distal fixation may increase bending and shear stresses. Other reported factors include varus alignment, high body weight, male sex, younger age, revision arthroplasty, modular stem design, corrosion, surface damage, and manufacturing defects. In a 2024 systematic review, Turnbull *et al.* found that most cases occurred in men and identified age below 63 years and body weight above 80 kg as significant risk factors [2].

We describe fracture of a cementless Zweymüller-type stem treated with an unconventional revision strategy. Because the distal fragment could not be safely removed, it was retained and combined with the proximal component of a modular ESOP short stem. Clinical and radiographic outcomes were assessed over 13 years.

## Case presentation

A 58-year-old man with no relevant medical history underwent primary cementless right THA with the DiaLoc system in 2008 for osteoarthritis. The perioperative course was uncomplicated, and full weight-bearing was permitted 5 weeks after surgery. He returned to full activity, including recreational ballroom dancing.

Serial radiographs subsequently demonstrated progressive stress shielding around the distal part of the femoral stem. As the patient remained pain-free, clinical and radiographic surveillance was continued.

In October 2013, he developed sudden severe thigh pain aggravated by hip flexion and rotation. Radiographs confirmed fracture of the femoral stem. Revision THA with removal of both fragments and implantation of a longer stem was planned.

During surgery, the proximal fragment was removed. Extraction of the distal fragment through a cortical window using a retrograde technique was unsuccessful. A Wagner-type proximal femoral osteotomy was considered, but the risks of additional bone loss, impaired osteotomy healing, and the need for a long revision stem were considered excessive. As the distal fragment was stable and well fixed, it was retained, and only the proximal component of a modular ESOP stem, size 15, was implanted. Fibrous tissue from the femoral canal was obtained for microbiological examination.

One unit of packed red blood cells was transfused because of intraoperative blood loss. Rehabilitation was introduced gradually. Transient postoperative fever was treated empirically with pefloxacin and cefotaxime. The wound healed normally, laboratory findings were unremarkable, and intraoperative cultures were negative. The patient was discharged on postoperative day 8, mobilizing with forearm crutches and remaining non-weight-bearing.

At 6 weeks, the wound was fully healed and the patient reported no significant pain; full weight-bearing was allowed. At 12 weeks, he had an almost complete range of hip motion, walked without assistive devices, and remained pain-free. Radiographs showed satisfactory implant position and progressive osseointegration. Subsequent examinations confirmed complete osseointegration, and the patient returned to work and physical activity.

In 2016, progressive osteoarthritis of the left hip was treated with primary cementless THA using the ESOP system, with a favorable outcome.

At 13 years after revision surgery, the patient remains asymptomatic and physically active, without pain or functional limitation. Radiographs show stable fixation, with no migration, osteolysis, or implant loosening.

## Discussion

Standard treatment of a fractured femoral stem involves removal of both fragments and implantation of a longer revision stem. Extraction of a well-fixed distal fragment may require an extended approach, cortical window, or extended trochanteric osteotomy. These procedures increase bone loss and may lead to intraoperative fracture, infection, non-union, and prolonged rehabilitation [3].

In the present case, failure to extract the distal fragment required modification of the original plan. Because the fragment was securely fixed and there was no evidence of loosening or periprosthetic joint infection, further surgical escalation was avoided. Implantation of the proximal component of a modular short stem provided stable reconstruction while preserving femoral bone stock.

The mechanism of failure was probably multifactorial. The patient was male, younger than 63 years at primary THA, and physically active. Progressive stress shielding indicated altered proximal load transfer, whereas firm distal fixation may have increased bending stresses at the transition between supported and unsupported portions of the stem. Repetitive cyclic loading may therefore have resulted in fatigue fracture.

Retention of a fractured stem fragment should not be regarded as routine treatment. It may be considered when the fragment is stable, infection has been excluded, satisfactory alignment, and fixation can be achieved, and removal would require a substantially more invasive procedure. Long-term clinical and radiographic surveillance is essential because experience with this reconstruction remains limited (Figs 1–5).

**Figure 1:**
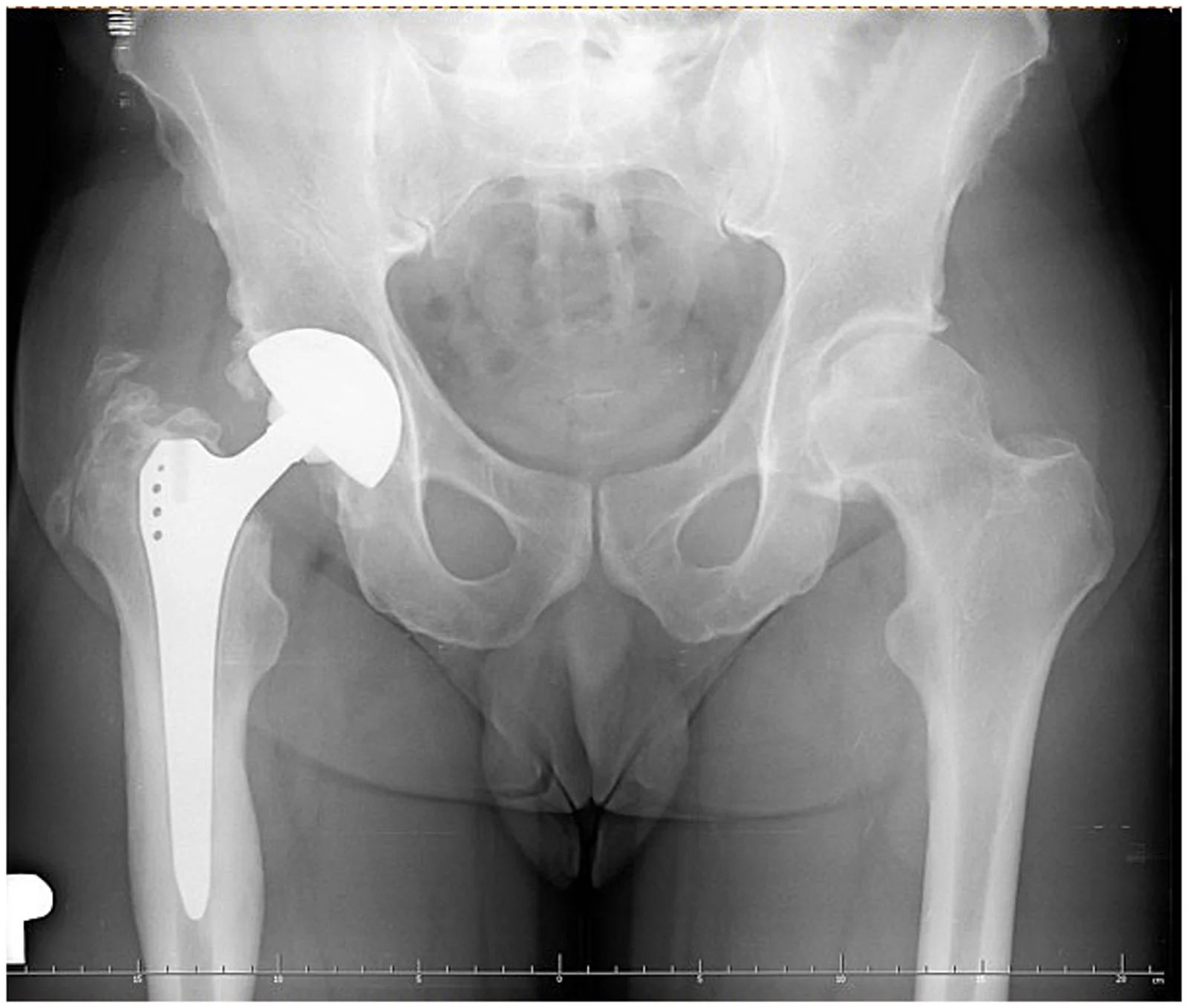
Patient following primary right total hip arthroplasty.

**Figure 2:**
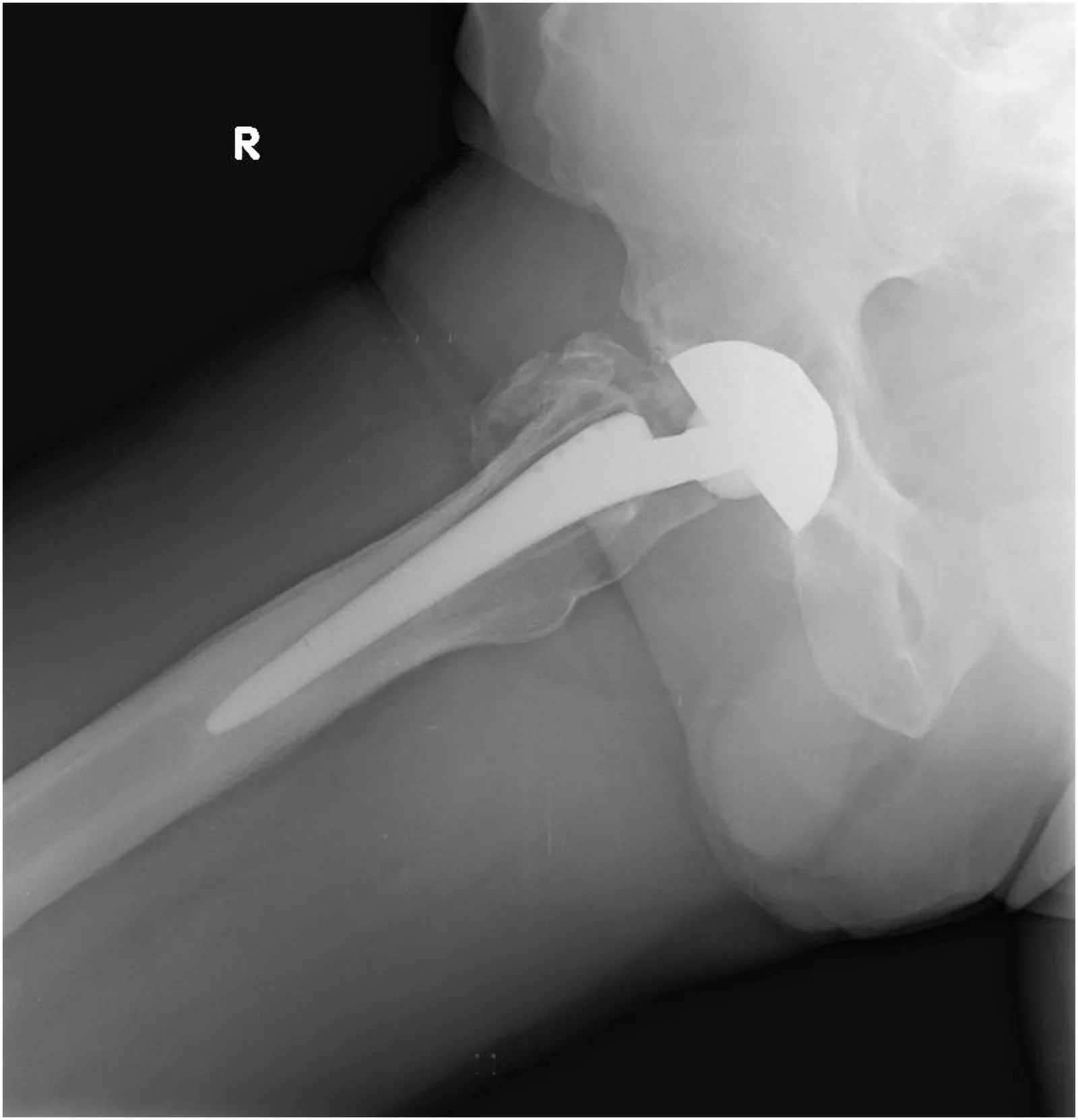
Patient at follow-up after the onset of pain. A fracture line is visible in the distal portion of the femoral stem.

**Figure 3:**
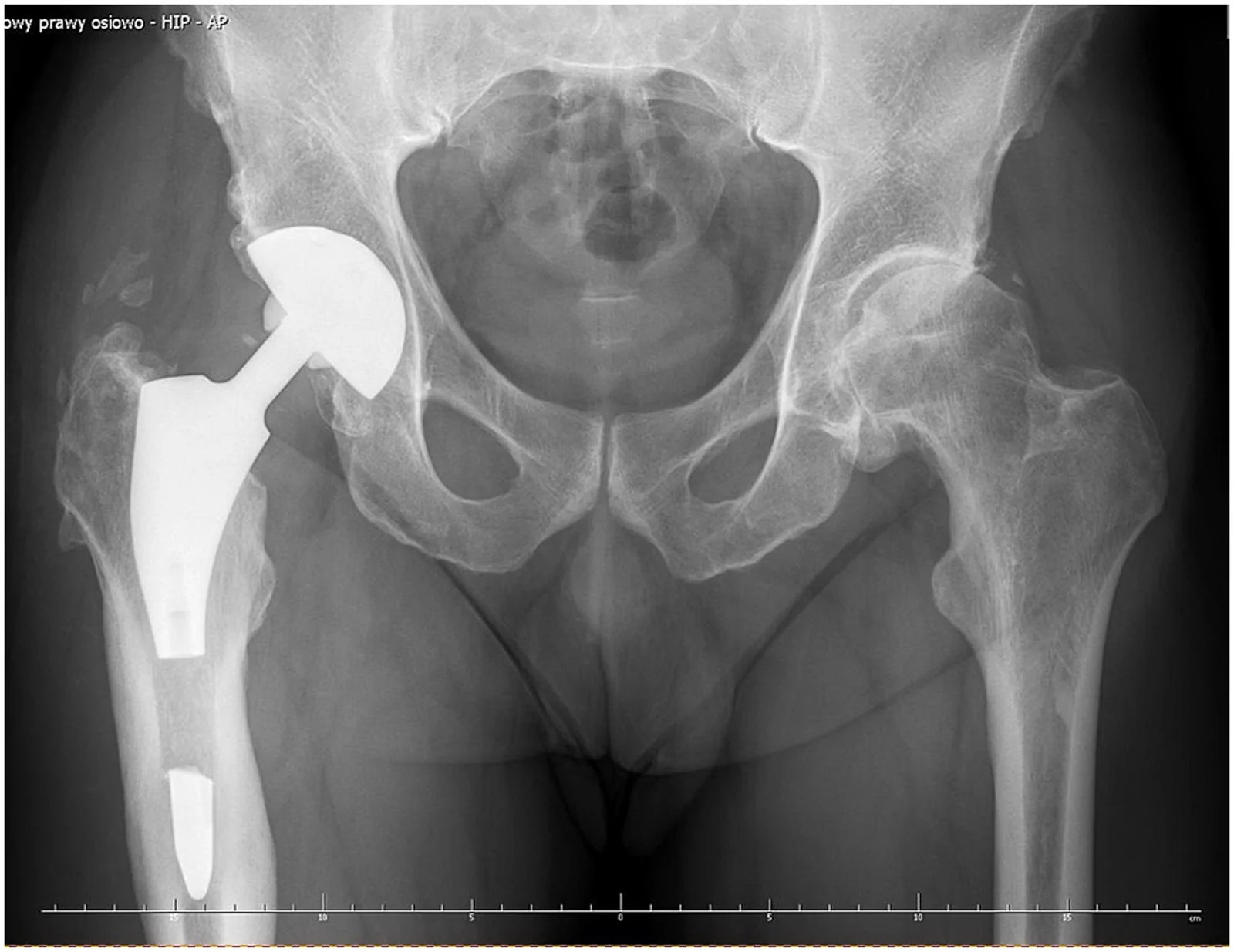
Radiographic follow-up after revision surgery.

**Figure 4:**
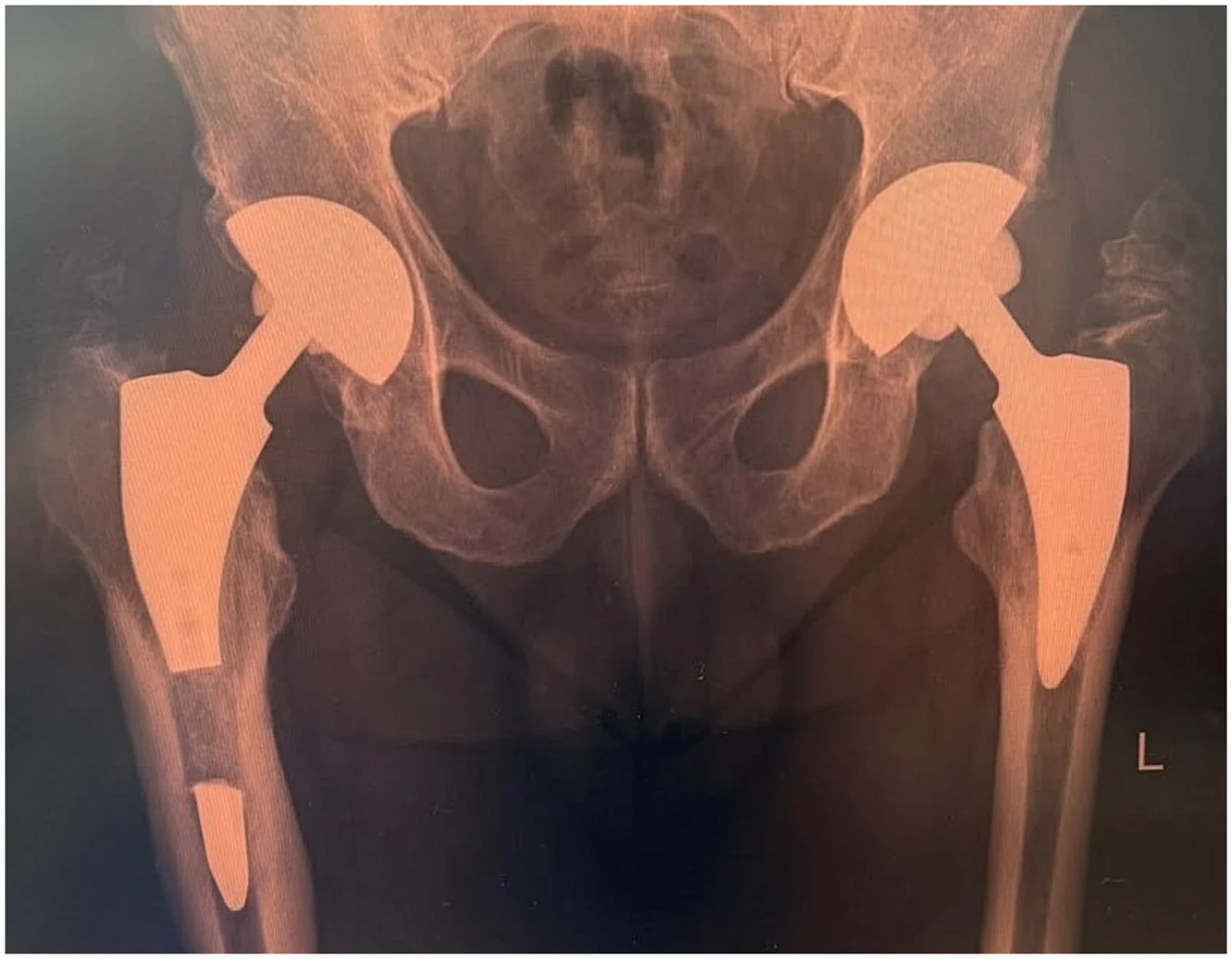
Patient 9 years after revision operation.

**Figure 5:**
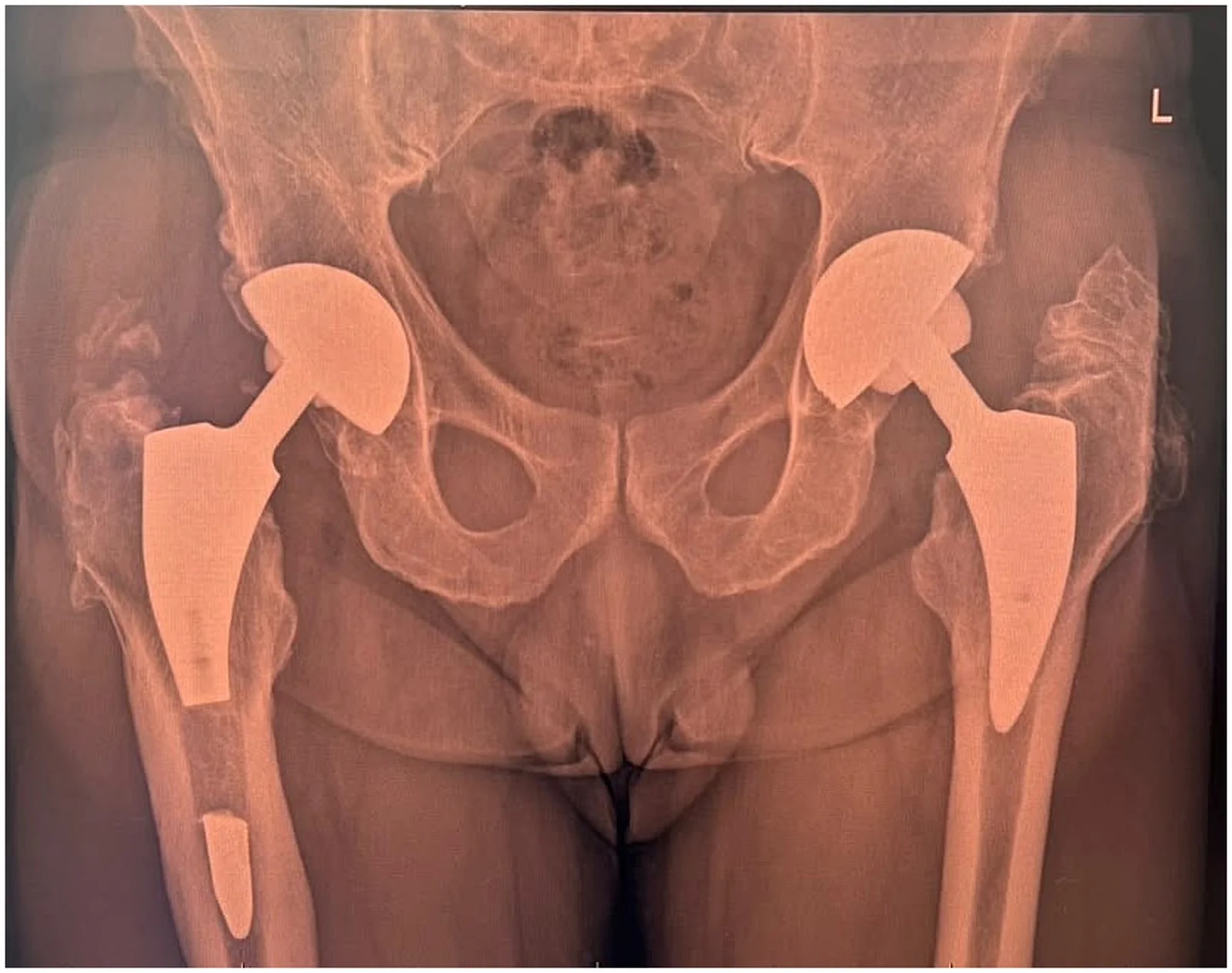
Patient 13 years after revision operation.

At the 13-year follow-up, the patient reported no pain or other complaints related to the operated hip. The patient achieved the maximum Oxford Hip Score at follow-up visits, indicating an excellent functional outcome.

## Conclusion

Retention of a well-fixed distal stem fragment combined with implantation of the proximal component of a modular ESOP short stem provided a durable result in this patient. At 13 years, he remains pain-free and fully functional, without radiographic evidence of loosening. Although conventional revision remains the preferred approach, individualized intraoperative decision-making may permit a less extensive reconstruction when removal of the distal fragment would expose the patient to disproportionate risk.
